# Virus Transfer from Personal Protective Equipment to Healthcare Employees’ Skin and Clothing

**DOI:** 10.3201/eid1408.080085

**Published:** 2008-08

**Authors:** Lisa Casanova, Edie Alfano-Sobsey, William A. Rutala, David J. Weber, Mark Sobsey

**Affiliations:** * University of North Carolina Chapel Hill, Chapel Hill, North Carolina, USA; † Wake County Human Services, Raleigh, North Carolina, USA

**Keywords:** PPE, occupational health, healthcare worker, contamination, dispatch

## Abstract

We evaluated a personal protective equipment removal protocol designed to minimize wearer contamination with pathogens. Following this protocol often resulted in virus transfer to hands and clothing. An altered protocol or other measures are needed to prevent healthcare worker contamination.

Caring for patients with communicable diseases places healthcare workers (HCWs) at risk. Infected HCWs may not only incur serious illness or death themselves but may spread infection to others. Methods to prevent HCW infections include vaccination (*1*), hand hygiene (*2*), and isolation of patients with communicable diseases (*3*).

A key aspect of patient isolation is proper use of personal protective equipment (PPE) to protect HCWs from pathogen exposure during patient care. PPE includes use of barriers (gowns, gloves, eye shields) and respiratory protection (masks, respirators) to protect mucous membranes, airways, skin, and clothing from contact with infectious agents (*3*). The importance of PPE was underscored in the recent outbreak of severe acute respiratory syndrome (SARS). HCWs accounted for ≈20% of cases (*4*); failure to properly use PPE was a risk factor for HCW infection (*5*).

This outbreak raised concern that HCWs could contaminate their skin or clothes with pathogens during PPE removal, resulting in accidental self-inoculation and virus spread to patients, other HCWs, or fomites. The Centers for Disease Control and Prevention (CDC) addressed this concern by designing a protocol to minimize contamination to the wearer during PPE removal (Figure 1) (*6*). However, the effectiveness of this protocol in preventing self-contamination has not been validated. To determine if removing PPE according to the CDC protocol prevents viral contamination of the wearer, a human challenge study was undertaken using a nonpathogenic virus.

**Figure 1:**
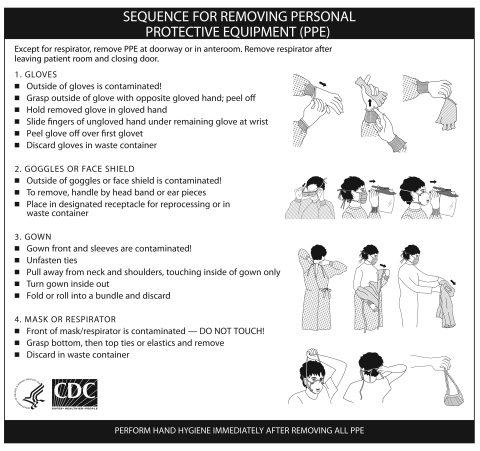
Centers for Disease Control and Prevention protocol for removing healthcare worker PPE.

## The Study

PPE (gowns, gloves, respirators, and goggles) donned by volunteers was contaminated with bacteriophage MS2, a nonenveloped, nonpathogenic RNA virus suspended in 0.01 mol/L phosphate-buffered saline and GloGerm (GloGerm, Moab, UT, USA), synthetic beads that fluoresce under UV light (for visual tracking of virus). Sites of contamination were as follows: front shoulder of gown, back shoulder of gown, right side of N95 respirator, upper right front of goggles, and palm of dominant hand. Each site was contaminated with a total of 10^4^ PFU of MS2 in 5 drops of 5 μL each. Participants performed a healthcare task (measuring blood pressure on a mannequin) and then removed PPE according to CDC protocol. Hands, items of PPE, and scrubs worn underneath were sampled for virus. Hands were sampled by using the glove juice method (*7*). Each hand was placed inside a bag containing 75 mL stripping solution (0.4 g KH_2_PO_4_, 10.1 g Na_2_HPO_4_, 1.0 mL Triton-X/L) and massaged for 60 seconds to cover all hand surfaces with solution. PPE items were immersed in 1.5% beef extract, pH 7.5, and agitated on a shaker for 20 minutes. Eluent from hands and PPE was assayed by the most probable number (MPN) enrichment infectivity assay (*8*). To prevent cross-contamination, samples from only 1 volunteer were processed at a time, and individual eluent samples were processed separately in a biological safety cabinet, with decontamination in between.

**Figure 2:**
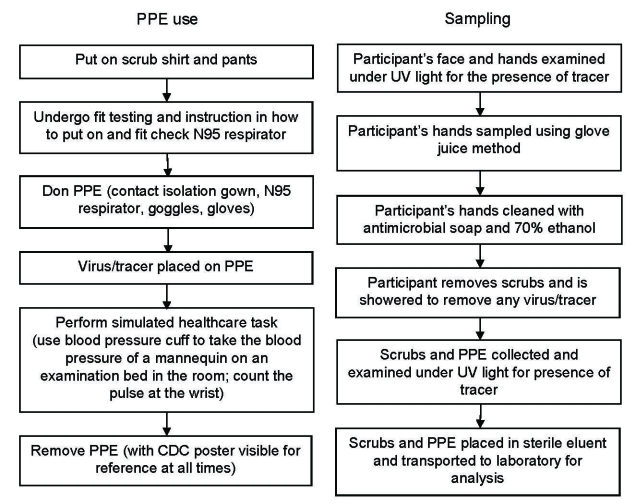
Protocol for human challenge experiments. PPE, personal protective equipment; CDC, Centers for Disease Control and Prevention.

When an a priori value of 25% was used for the 95% upper confidence limit when p (transfer) = 0, the sample size was N = 10. Protocols were approved by the University of North Carolina (UNC) Biomedical Institutional Review Board, and written informed consent was obtained. Enrolled participants met the following inclusion criteria: >18 years of age, nonpregnant, nonallergic to latex, no active skin disorders, and medical evaluation approval for N95 respirator fit testing and use (*9*). Experiments took place in a patient care room in the UNC Hospitals’ General Clinical Research Center. The experimental protocol is shown in Figure 2. Participants were shown the poster distributed by CDC (Figure 1) and given an opportunity to read it and ask questions. The poster was placed in front of the participants for reference while they donned and removed PPE.

Ten study participants were enrolled in this study: 9 women and 1 man. Nine participants were right-handed, and 1 was left-handed. Transfer of virus to both hands, the initially uncontaminated glove on the nondominant hand, and the scrub shirt and pants worn underneath the PPE was observed in most volunteers (Table). Because of the difficulty of sampling large facial areas, visible fluorescent tracer was used as the criterion to determine whether the face would be sampled. No tracer was observed on the facial areas of any volunteer. The fluorescent tracer was not a consistent indicator of virus contamination; virus was recovered both from sites where tracer was visible and where it was not detected.

**Table:** Frequency and levels of viral contamination of selected sites, virus transfer study, 2007*

Site	% Volunteers who transferred virus to site (N = 10)	Mean viral titer recovered from site (log_10_ MPN)*	% Contaminated sites with visible tracer (N = 10)
Nondominant glove	80	2.2	10
Right hand (skin)	90	2.4	20
Left hand (skin)	70	1.8	0
Scrub shirt	100	3.2	10
Scrub pants	75†	2.1	0
Face	0	–	–

*MPN, most probable number; –, not measured. †N = 8.

The amount of virus recovered was 1–3 log_10_ MPN for hands and 1–4 log_10_ MPN for scrubs. The mean amount of virus recovered from the right hand (the dominant hand of 90% of volunteers) was greater than that recovered from the left hand. While removal of gloves and gowns required 2 hands, mask and goggle removal was one-handed, which could have resulted in larger quantities of virus being transferred to the dominant hand during removal. In the single left-handed study participant, recovery of virus was greater from the left hand than the right (1.82 log_10_ vs. 0.98 log_10_ MPN). The mean amount of virus recovered from scrub shirts was significantly greater than that recovered from pants (p = 0.01), possibly because of contact with hands when the gown is pulled away from the shoulder during removal.

## Conclusions

PPE is vital for protecting HCWs from occupationally acquired infection during patient care, particularly from droplet- or airborne-transmitted diseases. However, removing PPE after patient care without contaminating skin or clothes is important. Although PPE is usually worn only for short periods, viruses such as influenza (*10*) and SARS coronavirus (*11*) can survive for hours on surfaces, and viral infection can be spread by surface-to-hand (*12*) and hand-to-hand contact (*13*).

Developing and validating an algorithm for removing PPE that prevents contamination of the skin and clothes of HCWs are key to interrupting nosocomial transmission of infectious agents. These experiments demonstrate that the current CDC algorithm is insufficient to protect HCWs from contamination during PPE removal. However, options that might prevent such contamination do exist, including double gloving, use of surgical protocols for PPE removal, and PPE impregnated with an antimicrobial agent.

A double-glove removal sequence would begin with removal of the outer glove, followed by removal of goggles or face shield, gown, and respirator/mask, and finishing with removal of the inner glove followed by hand hygiene; handling of PPE with ungloved hands is avoided. Borrowing PPE protocols from surgery, in which the ends of gown sleeves are tucked underneath gloves during wear, might also reduce contamination. When the HCW is finished, goggles and respirator are removed first, and gown and gloves are then removed together by peeling off both at the same time, again avoiding handling PPE with ungloved hands. Finally, the use of PPE impregnated with antimicrobial agents might also reduce or eliminate contamination of skin and clothes.

This study also indicates the need for continued emphasis on hand hygiene. A barrier to improving hand hygiene compliance rates is the belief that gloves make hand hygiene unnecessary (*14*). This is contradicted by our study and others showing that organisms can spread from gloves to hands after glove removal (*15*). Even if double gloving is incorporated into protocols for PPE use, it is not a substitute for proper hand hygiene. Before these or other candidate methods are introduced into clinical practice, their impact on the safety of HCWs should be validated by testing with methods such as we have described.
